# Tandem amino acid repeats in the green anole (*Anolis carolinensis*) and other squamates may have a role in increasing genetic variability

**DOI:** 10.1186/s12864-016-2430-y

**Published:** 2016-02-12

**Authors:** Riga Wu, Qingfeng Liu, Peng Zhang, Dan Liang

**Affiliations:** Key Laboratory of Gene Engineering of the Ministry of Education, State Key Laboratory of Biocontrol, School of Life Sciences, Sun Yat-Sen University, Guangzhou, People’s Republic of China

**Keywords:** Homopolymeric tract, Homopeptide, Squamates, Lepidosauria, Comparative genomics

## Abstract

**Background:**

Tandem amino acid repeats are characterised by the consecutive recurrence of a single amino acid. They exhibit high rates of length mutations in addition to point mutations and have been proposed to be involved in genetic plasticity. Squamate reptiles (lizards and snakes) diversify in both morphology and physiology. The underlying mechanism is yet to be understood. In a previous phylogenomic analysis of reptiles, the density of tandem repeats in an anole lizard diverged heavily from that of the other reptiles. To gain further insight into the tandem amino acid repeats in squamates, we analysed the repeat content in the green anole (*Anolis carolinensis*) proteome and compared the amino acid repeats in a large orthologous protein data set from six vertebrates (the Western clawed frog, the green anole, the Chinese softshell turtle, the zebra finch, mouse and human).

**Results:**

Our results revealed that the number of amino acid repeats in the green anole exceeded those found in the other five species studied. Species-only repeats were found in high proportion in the green anole but not in the other five species, suggesting that the green anole had gained many amino acid repeats in either the *Anolis* or the squamate lineage. Since the amino acid repeat containing genes in the green anole were highly enriched in genes related to transcription and development, an important family of developmental genes, i.e., the Hox family, was further studied in a wide collection of squamates. Abundant amino acid repeats were also observed, implying the general high tolerance of amino acid repeats in squamates. A particular enrichment of amino acid repeats was observed in the central class Hox genes that are known to be responsible for defining cervical to lumbar regions.

**Conclusions:**

Our study suggests that the abundant amino acid repeats in the green anole, and possibly in other squamates, may play a role in increasing the genetic variability, and contribute to the evolutionary diversity of this clade.

**Electronic supplementary material:**

The online version of this article (doi:10.1186/s12864-016-2430-y) contains supplementary material, which is available to authorized users.

## Background

Tandem amino acid repeats, also called homopolymeric tracts, are regions within protein featured by the consecutive occurrence of a single amino acid. They are translated from trinucleotide repeats that are generated mainly by slippage during DNA replication [1, 2]. Alternative mechanisms have also been proposed, such as unequal crossing-over during recombination [3–5]. Like other sequences, repeats will suffer mutations and indels, resulting in changes in the sequences. Due to the repetitive nature of the tracts, replication slippage is frequent [6]. Mispairing in the slipped-strand may be corrected by the cellular DNA mismatch repair system [7]. Under the equilibrium of replication slippage and mismatch correction, length polymorphisms of trinucleotide repeat are common, translating into contraction or expansion of amino acid repeats. Thus, tandem amino acid repeats are diverse in both sequence and length, being a potential source of genetic variability.

Tandem amino acid repeats are commonly found in eukaryotic proteins [8–10]. However, in different species, the frequency and size of amino acid repeats vary greatly [10–12]. It is reported that human and mouse have an amino acid repeat content higher than many other vertebrates such as chicken, frog and zebrafish. Human is also the species with the largest number of amino acid repeats among the vertebrates investigated so far [11, 13]. The various amino acid repeat contents may be related to the GC content bias of different genomes as several studies have reported a positive correlation between certain types of amino acid repeat content and GC content at the third codon position [14–17]. On the other hand, variations in replication slippage and the efficiency of the DNA mismatch repair system may also affect amino acid repeat frequency. In the proteomes, the distribution of amino acid repeats appears non-random. They are usually enriched in proteins related to transcription, DNA, RNA and protein binding, or development [2, 10, 13, 18–20]. Well-conserved amino acid repeats are found in greater abundance in highly constrained (Ka ≤ 0.02) proteins than in fast-evolving proteins in a comparison of human and mouse proteomes [21]. A significant association of amino acid repeats with alternatively spliced genes has also been observed [22]. Recently, Radó-Trilla et al. have reported that amino acid repeats were important driving forces of evolutionary changes in duplicated genes [23]. These suggest that at least some amino acid repeats are under selective constraints and may have important functions.

Within a protein, amino acid repeats are often embedded in disordered regions [24] that in normal conditions are unstructured, but can undergo a disorder-to-order transition upon binding with an interacting partner [25–27]. Amino acid repeats may thus get involved in or influence the protein-nucleic or protein–protein interactions [19, 28, 29]. Amino acid repeat expansions can be deleterious, causing neurological disorders and body abnormalities in human [30, 31]. However, changes of amino acid repeats can also be adaptive. For example, acquiring a polyalanine repressor domain in the insect Ultrabithorax protein (*Ubx*) may have facilitated the increased specialisation of the abdominal segments in insects [32]. In cetaceans, a novel expansion of a polyalanine stretch in *HoxD13* was supposed to contribute to the origin of flippers [33]. Another remarkable analysis demonstrated that length variation of amino acid repeats in developmental genes was associated with the great diversity of limb and skull morphologies observed in different dog breeds [34]. These indicate that in an evolutionary scenario, changes in amino acid repeats can contribute to the alterations of a species.

Squamate reptiles (lizards and snakes) are a versatile clade with diversity in many aspects of their biology, including body shapes, reproduction methods, venom production, etc. [35]. Lizards of the *Anolis* genus are some of the best examples in the study of adaptive radiation and convergent evolution [36], just like the terrestrial analogues to stickleback and cichlid fish [37]. The underlying mechanisms of their evolutionary agility are yet to be understood. In a phylogenomic analysis of reptile, a high density of tandem repeats has been revealed as a trait of the Bahamian green anole (*Anolis smaragdinus*) sequences, which diverges heavily from the repeat densities of the other reptiles [38]. Following this clue, we investigated in greater detail the repeats in the green anole proteome to find out whether the accumulation of tandem repeats has implications for their genetic plasticity. We focused on the tandem repeats in coding regions that would facilitate comparisons with other species and enable further function-related analyses. By carefully scrutinizing the green anole (*Anolis carolinensis*) proteome, 4953 amino acid tandem repeats were detected. After compared with other species in a large orthologous protein data set, the green anole exhibited the highest number of amino acid repeat containing proteins which were enriched in genes related to transcription and development. The green anole also possessed the greatest number and largest proportion of amino acid repeats that were not found in other species, indicating a high tolerance for amino acid repeat generation of the anole genome. Possible significances of the amino acid repeats were further studied by analysing an important family of transcriptional and developmental genes, the Hox family in a wide collection of squamate reptiles. Plentiful amino acid repeats were observed in the squamate Hox genes with a special enrichment in the central class Hox genes (paralogous groups (PG) 4–8) that are responsible for defining cervical to lumbar regions according to the collinearity of Hox clusters. Our study suggests that the abundant amino acid repeats may play a role in increasing the genetic variability of anoles, and possibly of other squamates as well.

## Methods

### Vertebrate sequences

The protein and cDNA (complementary DNA) sequences of the green anole (*Anolis carolinensis*, AnoCar2.0.68), the Western clawed frog (*Xenopus tropicalis*, JGI_4.2.68), the Chinese softshell turtle (*Pelodiscus sinensis*, PelSin_1.0.68), zebra finch (*Taeniopygia guttata*, taeGut3.2.4.68), mouse (*Mus musculus*, GRCm38.68) and human (*Homo sapiens*, GRCh37.68) were downloaded through Biomart at Ensembl using the AAstretch package [39]. These sequences were used for genome-scale amino acid repeat identification and repeat content comparison. For each gene the longest protein sequence was selected for further study.

For the intensive study of the amino acid repeats in the Hox gene family, Hox gene fragments were *de novo* sequenced from ten squamate species and one amphibian species (see below for details). In addition, Hox gene fragments of 17 other vertebrate species were downloaded from public resources. Table 1 listed in detail all the species and the usage of these species.

**Table 1:** The species used in this study

Taxonomy		Scientific name	Common name	Collection locality or source
Amphibia	Gymnophiona	*Ichthyophis bannanicus* ^a^	Banna caecilian	Beiliu, Guangxi, China
	Xenopodinae	*Xenopus tropicalis* ^b^	Western clawed frog	GenBank
Reptilia	Dibamidae	*Dibamus bourreti* ^a^	Bourret's blind skink	Hongkong, China
	Gekkonidae	*Hemidactylus bowringii* ^a^	House Gecko	Guangzhou, Guangdong, China
	Scincidae	*Plestiodon fasciatus* ^a^	Common Five-lined Skink	MCB249, gift from Matt Brandley
	Amphisbaenidae	*Amphisbaena caeca* ^a^	Puerto Rican worm lizard	MVZ Herps 232753
	Bipedidae	*Bipes biporus* ^a^	Five-toed worm lizard	MVZ Herps 236257
	Agamidae	*Calotes versicolor* ^a^	Garden lizard	Guangzhou, Guangdong, China
	Iguanidae	*Anolis carolinensis* ^b^	Green anole	GenBank
	Anguidae	*Anguis fragilis* ^a^	Slow worm	MVZ Herps 238523
	Varanidae	*Varanus salvator* ^a^	Water monitor	Jiangmen, Guangdong, China
	Elapidae	*Naja atra* ^a^	Chinese cobra	Shaoguan, Guangdong, China
	Typhlopidae	*Ramphotyphlops braminus* ^a^	Brahminy blind snake	Hongkong, China
	Galliformes	*Gallus gallusdomesticus*	Chicken	GenBank or Ensembl
	Estrildidae	*Taeniopygia guttata* ^b^	Zebra finch	GenBank
	Trionychidae	*Pelodiscus sinensis* ^b^	Chinese softshell turtle	GenBank
Mammal	Ornithorhynchidae	*Ornithorhynchus anatinus*	Duckbill platypus	GenBank or Ensembl
	Macropodidae	*Macropus eugenii*	Tammar wallaby	GenBank
	Didelphidae	*Monodelphis domestica*	Gray short-tailed opossum	GenBank
	Elephantidae	*Loxodonta Africana*	African bush elephant	GenBank
	Pteropodidae	*Pteropus vampyrus*	Large flying fox	GenBank
	Canidae	*Canis familiaris*	Dog	GenBank or Ensembl
	Bovidae	*Bos Taurus*	Cow	GenBank
	Leporidae	*Oryctolagus cuniculus*	Rabbit	GenBank
	Muridae	*Mus musculus* ^b^	Mouse	GenBank
	Leporidae	*Oryctolagus cuniculus*	Rabbit	GenBank
	Hominidae	*Homo sapiens* ^b^	Human	GenBank

GenBank or Ensembl means that Hox genes for these species were downloaded from these public databases

^a^ Hox gene fragments were *de novo* sequenced in this study, ^b^ species were also used in proteomic-scale analyses

### Identification of amino acid repeats

**Fig. 1:**
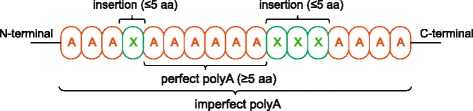
Schematic representation of a polyA repeat. A polyA repeat contains at least five consecutive A residues. The maximum length of residues other than A is five and the maximum proportion of this non-main residue is 30 %. If the proportion of main residue (A) is 100 % in a polyA repeat, it is thought to be a perfect repeat. Otherwise it is an imperfect repeat

The AAstretch package was able to scrutinise both perfect and imperfect amino acid repeats within protein. Since the polypeptides are commonly found imperfect (e.g., polyQs are imperfect in a number of polyQ-expansion diseases [40]), our settings allowed the insertions of different amino acid residues within a specific polypeptide. In our study, tandem amino acid repeats were defined as tracts with a minimum main residue content of over 70 %, and a core of at least five consecutive amino acid residue that can be extended N- and/or C-terminally with insertions of other residues of no more than five consecutive residues (Fig. 1). Thus, the identified amino acid repeats may have variable lengths (with a minimum of 5-residues) with main amino acid composition ranging from 70 to 100 % (100 % are perfect repeats, while others are imperfect repeats). Amino acid repeats were identified from the green anole proteome and an orthologous protein data set from six vertebrate species (Table 1). The orthologous protein data set was obtained through bidirectional BLASTs (Basic Local Alignment Search Tools) of the green anole proteome against those of the other species using the BLAST program [41]. Only the best bidirectional hits (the top reciprocal BLAST hits, E-value < 10^−20^) were considered as orthologous proteins. The green anole proteome and the orthologous protein data set from the six vertebrate species were scrutinised for amino acid repeats using the AAstretch package with the following parameters: the minimum consecutive repeat length was five; the minimum percentage of the main residue in an imperfect repeat was 70 %; the number of allowed insertions in a consecutive stretch of a single amino acid residue was five. It is important to note that amino acid repeat detection is still not well resolved yet with different detectors producing different inferences, reflecting characteristics of the underlying algorithms. It may depend on the degree of divergence in amino acid repeat, and repeat features such as the length of repeat sequence as well as the minimal repeat unit [42].

We extracted information of the amino acid repeats, including the amino acid repeat sequence, the DNA sequence encoding the repeat, repeat size, major amino acid type and position within the protein, and calculated the proportion of the longest consecutive pure codon run size to the complete repeat size and named this value as PLP (proportion of the longest consecutive pure codon to the complete repeat size) [21].

### GC content comparison

For each species, GC contents of all the orthologous coding regions and of the coding regions of the repeat containing proteins after removing the repeats were compared.

### Gene Ontology (GO) annotation

GO terms were extracted simultaneously as the protein sequences were downloaded from Ensembl using the AAstretch package. Functional classification of the orthologous proteins was done following the strategy in [43] with some modifications. By grouping together the terms that were functionally related and tended to co-occur in the same protein we created four nonredundant functional groups. The first group, “Transcription factor and/or Development”, identified through key words “transcription”, “development”, “genesis” and “differentiation”. The second group, “Signal”, contained signal transduction and transport related functions, identifying through “signal transduction”, “receptor”, “response”, “transport” and “membrane”. The third group, “Metabolism”, identified through “metabolic”, “transferase”, “catabolic”, “biosynthetic”, “protein translation/modification/folding” and “proteolysis”. All the remaining proteins, including those not classified into the above groups and those without clarified annotations, were classified as the fourth group, “Others”. To acquire non-overlapping data sets, genes already classified into a previous group(s) were excluded from the rest. This classification was based on the human GO terms and the orthologous genes in other species were supposed to have similar functions.

### Amino acid repeat comparison in orthologous sequences

We determined whether amino acid repeats appearing in the orthologous protein of one species were also present in the other species. Orthologous protein sequences were aligned using PRANK’s (The Probabilistic Alignment Kit) codon-based alignment with default settings [44]. The identified amino acid repeat sequences in the six species were mapped onto the alignments to look for conserved repeats among different species. Conserved repeats refer to repeats formed by the same amino acid type located in an equivalent position, that is, overlapped by at least one codon in the orthologous protein alignment. Repeated DNA sequences (e.g., GGCGGCGGCGGC) translated into two different frames in two species (e.g., as polyP or polyA) were not considered as conserved repeats.

### Sequencing of Hox gene fragments

Hox gene fragments were *de novo* sequenced from ten squamates and one amphibian (Table 1). This study was performed in strict accordance with the guidelines developed by the China Council on Animal Care and Use. All animal processing procedures were approved by the Institutional Animal Care and Use Committee of Sun Yat-Sen University (permit number: 2011-025).

Total genomic DNAs were extracted from the tissues (liver, muscle or skin) preserved in ethanol using the standard salt extraction protocol. In addition to the primers from our previous publication [45], new degenerate primers (Additional file 1) were designed according to the following strategy to obtain as many exon one (composing most of the non-homeodomain region) sequences as possible. Hox exon one together with the flanking sequences (i.e., the untranslated regions and introns) of Menado coelacanth (*Latimeria menadoensis*), the Western clawed frog, zebra finch, the green anole, mouse and human were collected from GenBank, Ensembl or UCSC (University of California, Santa Cruz) Genome Browser and aligned with BioEdit [46]. Degenerate primers were firstly designed in the flanking noncoding regions that were conserved from coelacanth to human or conserved from frog to human. If the primers in noncoding regions didn’t work, we designed degenerate primers in coding regions. For genes that were still difficult to amplify, more than one set and different combination of primers were used for semi-nested PCR to increase the probability of successful amplification and the reverse primer of the first step might locate in the homeodomain regions (exon two). PCR with genomic DNA was performed in 25 μl reaction volume with TransT Taq DNA polymerase (TransGen, Beijing). The cycling parameters were as follows: an initial denaturation step at 94 °C for 2 min, 45 cycles of 94 °C for 30 s, 45–55 °C for 1 min, 72 °C for 30 s, and a final extension step at 72 °C for 10 min. For nested PCR, the second step PCR was conducted with the same procedure adding 1 μl of the first step PCR product as template. PCR products were then purified by agarose gel extraction (Tiangen, Beijing) and cloned into an in-house T vector. Positive recombinant clones were identified by colony PCR and the PCR products were cleaned with ExoSap treatment and sequenced on an automated ABI3730 DNA sequencer. All sequences were examined by BLAST search against GenBank to confirm they were our target genes but not the paralogous genes. Single gene phylogenetic trees were also made with our data to detect conflicts with the common species tree. If a sequence hit its target gene in the BLAST search and its position on a phylogenetic tree was as expected, we considered it as a target gene.

### Identification of amino acid repeats in the Hox genes

**Fig. 2:**
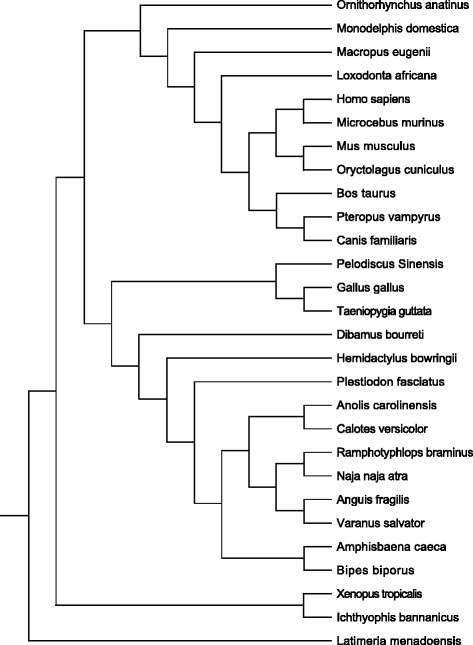
The commonly accepted relationship of the studied species based on Time tree of life [64]

In addition to our *de novo* sequenced squamate and amphibian sequences, Hox gene fragments of other vertebrate species were downloaded from public resources (see Table 1 for detailed information). A phylogenetic tree of the studied species was shown in Fig. 2. BioEdit was used to view, edit and translate the sequences. Alignments were performed using PRANK’s codon-based alignment with default settings. Protein alignments were generated according to nucleotide alignments. We identified amino acid repeats of size five or longer in each Hox gene using the AAstretch program with the same parameter settings as used in the proteomic analysis. Based on amino acid repeat type and their location in the alignment, the amino acid repeats were classified into two categories: repeats appearing in all the studied species in a clade were termed clade-common repeats, and those appearing only in some of the studied species in a clade were named non-clade-common repeats.

## Results

### Extensive accumulation of amino acid repeats in the green anole proteome

We scrutinised the green anole proteome for amino acid repeats of size 5 or longer with the AAstretch program (see Methods for parameter settings). A total of 4953 amino acid repeats was identified in 3273 proteins (constituting 18.7 % of the proteome, see Additional file 2 for the list of the amino acid repeats). All 20 amino acids but W (tryptophan) were repeated in the green anole at different sequence lengths. The three most commonly found amino acid repeat types were E (glutamic acid), S (serine), P (proline) (see Table 2 and Additional file 3 for the others and details).

**Table 2:** Statistics of the commonly found amino acid repeat types in the green anole proteome

AA type	Count	Average length (Maximum length)	Average length of consecutive AA tract (Maximum length)	Average PLP	Number of PLP = 1 (Percentage)	Number of Codons PLP = 1
E	1004	11 (98)	6.7 (30)	0.41	49 (4.9 %)	GAG (34), GAA (15)
S	792	10.2 (78)	6.4 (25)	0.35	31 (3.9 %)	AGC (16), TCC (8), AGT (4), TCG (1), TCA (1), TCT (1)
P	686	10.9 (61)	6.7 (20)	0.31	13 (1.9 %)	CCT (6), CCC (4), CCA (2), CCG (1)
G	566	11.4 (64)	6.8 (24)	0.39	15 (2.7 %)	GGA (10), GGC (4), GGG (1), GGT (0)
A	392	9 (30)	6.5 (15)	0.44	33 (8.4 %)	GCC (14), GCG (8), GCA (7), GCT (4)
L	361	8.2 (29)	5.9 (18)	0.37	14 (3.9 %)	TTA (6), CTC (5), CTG (2), CTT (1)
Q	303	10.4 (103)	7.1 (48)	0.51	40 (13.2 %)	CAG (37), CAA (3)
K	242	8.8 (28)	5.8 (11)	0.42	13 (5.4 %)	AAG (10), AAA (3)
D	138	8.3 (25)	6.1 (20)	0.53	23 (16.7 %)	GAT (20), GAC (3)

All lengths in this table refer to number of amino acid. AA means amino acid. PLP is the proportion of the longest consecutive pure codon run size to the complete repeat size

The length of the amino acid repeats varied greatly which appeared independent of the length of the protein it lay in (data not shown). As repeat size increased, the number of repeats decreased sharply (Additional file 4). 98.8 % of the amino acid repeats found in the green anole were less than size 30 with uninterrupted homopolymeric tract less than 24 amino acids. Several amino acid types, such as E, S, P, G and Q, can form repeats longer than 30 residues with the longest one reaching 103 amino acids (Table 2). Correspondingly, the average repeat lengths for repeat types E, S, P, G and Q were all greater than 10, longer than the others (Table 2). The uninterrupted homopolymeric tracts within these long repeats were mostly less than 30 except two polyQ tracts expanding to 47 and 48 residues. They are located in two sequence-specific DNA binding transcription factors, the clock circadian regulator and forkhead box P2 (FoxP2), respectively. FoxP2 protein is known to have a large polyglutamine tract in many vertebrates. Nevertheless, the one in the green anole lizard is the longest ever studied. Similarly, the polyglutamine tract in the clock circadian regulator of the green anole lizard is far longer than those observed in other vertebrates (mostly less than 10 consecutive glutamines). The lengths of these two polyQ tracts are much beyond the normal range. Whether they have any impacts on the protein functions is worth further investigation.

For each repeat we calculated the proportion of the longest pure codon run size to the complete repeat size and named this value as PLP [21]. PLP = 1 meant that the repeat was encoded completely by pure codon runs. Though reverse mutations could turn an impure codon run back into a pure one, it happened at a low frequency especially for long repeats. Thus repeats with PLP = 1 were mostly considered as young repeats. The average PLP and the percentage of repeats encoded by pure codon runs (PLP = 1) were shown in Table 2 for each repeat type. Some amino acids with the same codon degeneracy, for example, E and D, had quite different average PLP. The proportions of PLP = 1 repeats in D and Q were much larger than in other amino acid types. Within each amino acid, the number of PLP = 1 repeats varied for different codons (Table 2).

Finally, the spatial distribution of the repeats in the corresponding proteins was calculated, which might be biologically important as repeats located at either the amino terminal or carboxyl terminal were involved in many diseases [47]. The strongest bias of locating at the N-terminal of proteins was seen in Leucine (L) repeats, which have been reported to be enriched in membrane proteins [43] and possibly function as signal peptides [11]. Besides, G, A and S repeats also showed a localization bias to the amino terminal of the proteins (Additional file 5).

### Comparison of the amino acid repeat content of the green anole with other vertebrates

To inspect the anole’s amino acid repeats in an evolutionary context, we retrieved a set of 7682 orthologous proteins from the proteomes of six different vertebrate species (the Western clawed frog, the green anole, the Chinese softshell turtle, the zebra finch, mouse and human), and screened for tandem amino acid repeats. Remarkably, the green anole contains the largest number of amino acid repeats in total (2413), even more than the two mammals, human (2393) and mouse (2225), and 65 and 56 % greater than the other two sauropsid species, the Chinese softshell turtle (1462) and the zebra finch (1550), respectively (see Additional files 6, 7, 8, 9, 10 and 11 for the amino acid repeat list of each species). The Western clawed frog had the smallest number of amino acid repeats among the six vertebrates with only 1218 repeats in total. The most commonly found amino acid repeats in the six species are shown in Additional file 12 where the green anole exhibited a pattern closer to the Chinese soft shell turtle and the zebra finch but anole outnumbered the latter two in every repeat type. When compared with the human and mouse proteomes, the green anole proteome had more E, G, Q and S repeats, but less A, L and P repeats. Several residues that rarely appeared in amino acid repeats in other species were found to form repeats in the green anole (see Additional file 13).

**Fig. 3:**
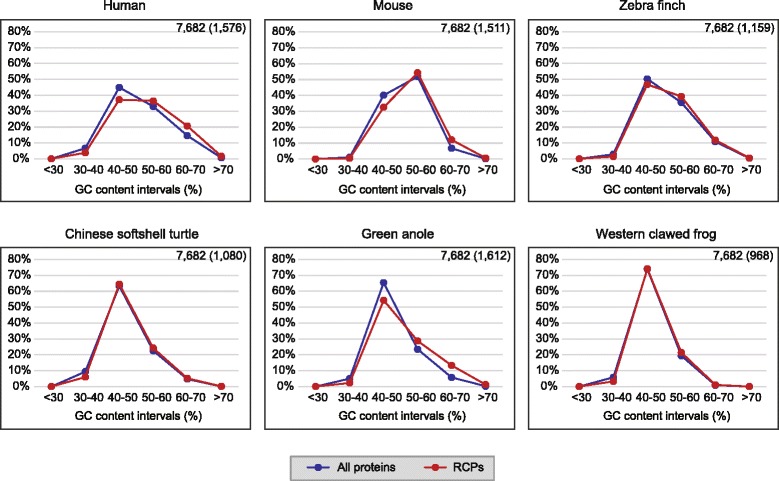
Percentage distribution of coding sequences in different GC content ratio intervals. “All proteins” refers to coding regions of the complete orthologous protein data set, and “RCPs” refers to coding regions of repeat containing proteins after discarding the regions encoding repeats. The numbers on the upper right corner of each plot indicate the total number of the orthologous proteins and the number of the RCPs in the species (*in parentheses*)

Since repeat occurrence might be related to GC richness, we analysed the GC content in the coding regions of the repeat-containing proteins (RCPs) and that of the complete orthologous data set (Fig. 3). In the complete orthologous data set, all the species peaked at the GC content interval of 40–50 % except mouse that peaked at the GC content interval of 50–60 %. While the distributions of GC content in RCPs of the Chinese softshell turtle and the Western clawed frog were almost the same as that in the complete orthologous data set, there were displacements, from slight to obvious, towards higher GC content in the RCPs of the zebra finch, mouse, the green anole and human. Thus, it appeared that GC richness was related to the high amino acid repeat content of the green anole, human and mouse in the orthologous data set.

**Fig. 4:**
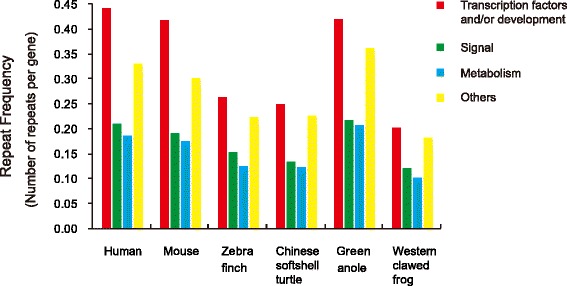
Frequency of amino acid repeats in proteins from different functional groups. The frequency (*y-axis*) of repeats was calculated through dividing the number of amino acid repeats within a specific functional group with the number of orthologous proteins of that group. The functional groups were defined from Gene Ontology annotations (see Methods)

The orthologous proteins in the six vertebrates were then grouped to four functional groups (“Transcription factor and/or Development”, “Signal”, “Metabolism” and “Others”) according to GO terms. Repeat frequencies (the ratio of the number of amino acid repeats within a group to the number of orthologous proteins in the same group) were calculated for each functional group. We detected the greatest number of amino acid repeats in the group “Transcription factor and/or Development” in all species studied, consistent with the enrichments observed in previous analyses [2, 10, 13, 19, 20]. For each functional group, the repeat frequency in the green anole was more similar to that in human and mouse, much higher than the other three non-mammal species (Fig. 4). In all the six species, “Transcription factor and/or Development” related proteins specifically preferred Q, H and A repeats and excluded R, K, L and E repeats. “Signal” related proteins preferred L and R repeats and excluded A, Q and G repeats. “Metabolism” related proteins favoured K repeats but tended to exclude H, Q, S and P repeats (Additional file 14).

Next we investigated to what extent the amino acid repeats in the green anole were conserved in other vertebrates. Pairwise comparisons of the six species were performed to detect conserved repeats which we defined as repeats of the same amino acid type located in an equivalent position of orthologous proteins. Of the 2413 anole amino acid repeats, about half were also present in at least one other species, which we temporally called the conserved group. The fraction of anole amino acid repeats found in other species ranged from 36.2 % (874) in human to 20.5 % (495) in frog, and 274 of the anole repeats appeared in all other five species. Besides the conserved repeats, there remained 1201 repeats found only in anole, which we temporally called the anole-only group. The conserved and anole-only groups of amino acid repeats had quite different features. The average length of the conserved repeats was longer than that of the anole-only ones (*p*-value < 0.01, u-test; Table 3). Amino acid repeats with size longer than 30 residues in anole mainly (35/40) fell into the conserved groups. They were longer than the corresponding repeats in the other species (*p*-value < 0.005, the Mann-Whitney-Wilcoxon test), suggesting expansions in the green anole. On the other hand, the anole-only group contained most of the repeats encoded by pure codons (PLP = 1), and had an average PLP much higher than the conserved groups. This implied that the anole-only repeats were relatively young, likely appearing in the lineage of anoles or the lineage of squamates. The pairwise analyses were also applied to the other five species. When calculating species-only repeats for human, mouse, zebra finch and the Chinese soft shell turtle, we excluded the close-related species (i.e., mouse is the close relative of human and the Chinese soft shell turtle is the close relative of zebra finch) because the presence of close-related species would lower the number of species-only repeats for these four species. The results showed that anole had the highest number and largest proportion of species-only amino acid repeats among the six species studied (*p*-value < 0.01, the Fisher exact test for all pairwise comparisons of the green anole with the other 5 species).

**Table 3:** Basic information of the amino acid repeats in different conservation groups from the green anole

Data set	Count	Average length (Maximum length)	Average length of consecutive AA tract (Maximum length)	Average PLP	Numcer of PLP = 1 (Percentage)
All repeats	2413	10.1 (103)	6.5 (48)	39.6	130 (5.4 %)
Anole-only repeats	1201	9.0 (47)	6.3 (31)	45.8	102 (8.5 %)
Repeats conserved in all 5 species	274	11.0 (103)	6.4 (48)	31.6	4 (1.5 %)
Repeats conserved in less than 5 species	938	11.1 (88)	6.7 (47)	34.1	24 (2.6 %)

### High content of amino acid repeats in the Hox genes of squamates

**Fig. 5:**
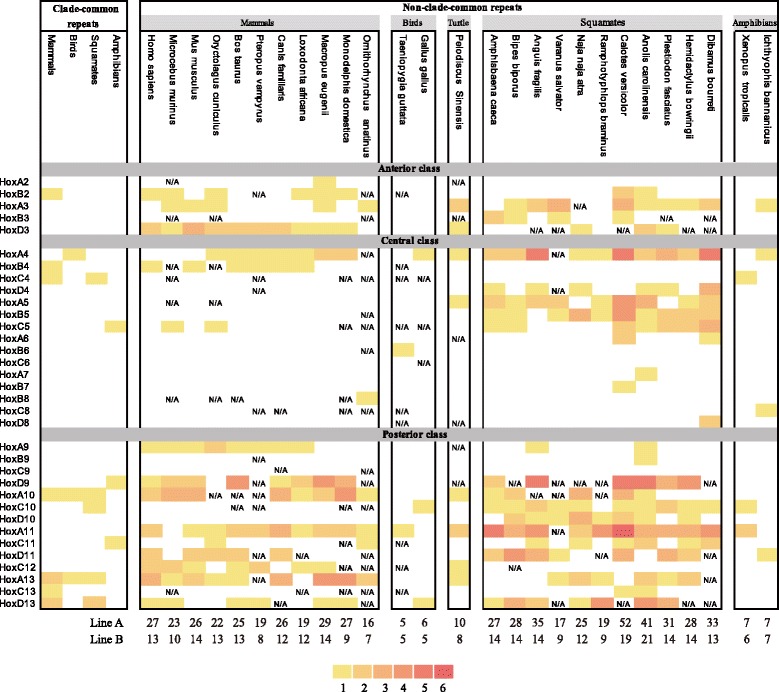
Amino acid repeat enrichment in the Hox family. For all the analysed species both clade-common repeats (those appeared in all species in a clade) and non-clade-common repeats (those appeared in only a fraction of the species in a clade) are shown. The clade-common repeat for turtle is not shown because only one species (*Pelodiscus Sinensis*) of this clade is studied. Note that only 34 Hox genes were successfully isolated from the squamate reptiles and compared with other species. The Hox genes are classified as the anterior class, the central class and the posterior class following traditional criterion. The colour depth increases as the number of repeats increases and the numbers of repeat are given beneath the corresponding colour blocks at the bottom of the figure. The total numbers of amino acid repeats in the shown Hox genes per species (LineA) and the total numbers of amino acid repeat containing Hox genes per species (LineB) are given under the chart. “N/A” means that the sequence is currently unavailable

To verify whether the high content of amino acid repeats was a clade-common feature and to look for possible significance of the amino acid repeats, we extended our analyses into a wide collection of squamate species. Without the sequenced genomes, we focused our study on an important family of transcriptional and developmental genes—the Hox genes. In the green anole proteome analysis above, we noticed that the green anole’s Hox family had accumulated an especially high content of amino acid repeats. Of the 37 Hox genes retrieved from the green anole genome, 23 genes (62 %) contained at least one stretch of amino acid repeat, highest among the six species studied (human 15, mouse 14, zebra finch 6, the Chinese softshell turtle 8 and frog 6). Since all the identified amino acid repeats are located in the non-homeodomain regions, we amplified the first exons of the Hox genes (composing most of the non-homeodomain region) from ten other squamate species that cover a wide taxonomic range (Table 1). Fragments of 34 Hox genes were successfully isolated. Most of them covered more than 90 % of the first exons except for the *HoxD8* fragments that were the C-terminal part of the first exon. With the same method, we also obtained the first exons of Hox genes from the basal amphibian, the Banna caecilian (*Ichthyophis bannanicus*) [48–50]. For comparison, data for other vertebrates were collected from public resources (Table 1). We specifically included 11 mammal species over a wide taxonomic range to form a group with the same number of species as the squamates (see Additional file 15 for the gene identifiers of all the analysed Hox genes). The analyses on the 34 Hox genes showed that the number of Hox genes containing repeats varied considerably from one species to another. Comparing the two groups, significantly more Hox genes in squamates had repeat(s) than in mammals (*p*-value < 0.03, the Mann-Whitney-Wilcoxon test). On average, 15 (44 %) of the 34 Hox genes in squamates had at least one repeat, while the averages observed for mammals, birds, turtles and amphibians were 13, 6, 8 and 6 genes, respectively (see details in Fig. 5 for the enrichment of amino acid repeats in each species and Additional file 16 for the list of amino acid repeats identified in the Hox family). Thus, high contents of amino acid repeats in Hox genes were quite common in squamate reptiles.

To further compare the two repeat-rich clades, i.e., squamates and mammals, their amino acid repeats were classified into two categories: clade-common repeats (those appeared in all the species studied in a clade) and non-clade-common repeats (those appeared in only a fraction of the species studied in a clade). Two clade-common amino acid repeats were conserved in squamates and mammals, which were a polyG tract in HoxA10 and a polyA tract in HoxA13. Expansion of this polyA tract in HoxA13 was shown to be a partial cause of the hand-foot-genital syndrome in human [51]. Besides, four clade-common amino acid repeats were identified in squamates, distributed in three Hox genes (polyP in *HoxC4*, polyP in *HoxC10*, polyS and polyA in *HoxD13*) while seven clade-common amino acid repeats were found in six mammal Hox genes (polyP in *HoxB2*, polyP in *HoxB4*, polyP in *HoxC4*, polyA in *HoxA13*, polyG in *HoxC13*, polyA and polyS in *HoxD13*). Among them, only the function of mammalian clade-common polyA tract in *HoxD13* has been documented [52, 53]. This polyA tract is also found in chicken, but absent from squamate reptiles. The clade-common polyA tract of squamates is located in a different region of *HoxD13*. Greater differences of the two clades, however, lay in the non-clade-common repeats (Fig. 5). 80.3 % of the amino acid repeats found in the squamate lineage were non-clade-common, the number of which was 1.7-fold higher than that in mammals. While the posterior class Hox genes (PG9 to PG13) of both clades have plenty of repeats, a sharp difference exists in the central class Hox genes (PG4 to PG8), especially PG5, that the total number of non-clade-common repeats in the central class Hox genes of squamates was 5-fold higher than that of mammals (91 vs. 18). It was less likely related to GC content as the average GC content of the central class Hox genes in squamates was lower than that in mammals, and was also lower than that of the anterior and posterior classes of Hox genes in squamates.

Overall, the Hox genes that contained five or more repeats in squamates were *HoxA3*, *HoxA4*, *HoxA5*, *HoxB5*, *HoxD9*, *HoxA10*, *HoxA11, HoxA13* and *HoxD13*, while those in mammals were *HoxD9*, *HoxA10* and *HoxA13*. For both squamate and mammal Hox genes, the most frequent amino acid repeat types were A, G, P and S (accounting for 90.4 % of the total repeats).

## Discussion

Squamates are the most diverse reptile group, with approximately 7400 living species. They have adapted to a variety of environments indicating a wealth of physiological and morphological diversity [36]. The genome sequence of *Anolis carolinensis*, a squamate representative, allows us to explore the underlying genetic plasticity and get a better understanding of amniotes evolution. In this study, we focused on tandem repeats in coding regions and identified an abundance of amino acid repeats in the green anole proteome. The comparative analysis in a large data set of orthologous proteins from six vertebrate species further revealed that the green anole possessed an amino acid repeat content comparable to mammals and much higher than the other two sauropsids and the amphibian. The functional group distribution of the amino acid repeats in the green anole was also more similar to human and mouse but divergent from the other three non-mammal species. Our results corroborated with the general pattern of tandem repeats observed in a phylogenomic analysis of the reptile genomic clones [38]. Besides, our data also add to the knowledge about the amino acid repeat inventory of vertebrates and demonstrate that high amino acid repeat content is found outside mammals in cold-blooded species such as lizards. Also, the sequencing and analyses of Hox gene fragments from 10 other squamate species revealed many amino acid repeats, implying that squamates might generally show high tolerance for amino acid repeat occurrence and maintenance.

From the observation that the green anole and the mammals had a large number of tandem repeats while the zebra finch and the Chinese soft shell turtle had much fewer, it was hypothesised that the common amniotes ancestor had a repeat-rich genome, then massive loss of repeats occurred in the lineages of turtles and birds. Alternatively, the scenario might be more complex, involving independent gains and reductions of repetitive elements in different lineages [38]. Our pairwise comparison showed that the green anole, the zebra finch and the Chinese softshell turtle had similar numbers of amino acid repeats conserved in mammals, though the green anole possessed many more amino acid repeats in the orthologous protein data set than the zebra finch and the Chinese softshell turtle. That is, though the number of amino acid repeats in green anole was comparable to those in mammals, only a small fraction of the amino acid repeats were conserved between the green anole and mammals. On the other hand, the green anole had the greatest number of species-only amino acid repeats and most of the anole pure repeats (PLP = 1) belonged to the anole-only group. These results favoured independent gains and reductions of amino acid repeats in different lineages, and suggested that green anole had a large number of amino acid repeats not because it retained significantly more ancestral repeats but because it gained more new ones subsequently, either in the *Anolis* lineage or in the squamate lineage. In contrast, zebra finch and the Chinese softshell turtle gained far fewer, resulting in smaller numbers of amino acid repeats observed. Thus it seems that the anole genome could put up with the frequent occurrence of new tandem amino acid repeats without fatal effects and showed a high tolerance to these low-complexity sequences during evolution. Why the anole genome has more amino acid repeats than the other reptiles is intriguing but remains unknown. One possible reason may lie in high GC content, as our result showed that the species with high amino acid repeat contents, i.e., green anole, human and mouse, all have relatively higher GC contents. Besides, further study may probe into the efficiency of the DNA mismatch repair system in anoles. It has been reported that cells or organisms with DNA mismatch repair system deficiency exhibited a significant increase in frequency of microsatellite instability [54–56].

It is noteworthy that the identification of amino acid repeats relies greatly on the quality of the studied genomes. Trinucleotide repeat sequences encoding amino acid repeats are usually low complexity sequences. If genome sequencing was of low quality, these low complexity repeat regions might not be properly sequenced or assembled, which would result in underestimation of amino acid repeat content. Moreover sequencing errors would introduce false mutations into the codon runs, leading to underestimation of the amino acid content especially the number and length of pure repeat runs. It is possible that the amino acid repeat contents in the Western clawed frog, the green anole, the Chinese softshell turtle and the zebra finch were less precise than the well-sequenced human and mouse. The comparisons of amino acid repeats among the six species thus were preliminary. As the sequencing technology and assembling algorithm improved, future study will provide results closer to the truth.

The amino acid repeats containing genes in the green anole have an apparent stronger enrichment in genes related to transcription and development. In an important family of developmental genes, the Hox gene family, the green anole has the highest number of amino acid repeats, even higher than human and mouse. Hox genes are well known for their function in defining body plan during embryo development [57] and are usually under stringent evolutionary constraints. However, considering there were multiple transposable elements in the intergenic regions [58] and many amino acid repeats in the coding sequences (this study), the constraints on the green anole Hox clusters seemed to have been altered. The transposable elements in intergenic regions might influence the regulatory elements and the amino acid repeats in coding sequences might affect the protein structures and activities. Together, they could lead to different interpretations of the Hox code in green anole. Further sequencing of the Hox gene fragments from 10 other squamates indicated that the accumulation of amino acid repeats in Hox genes appeared a general feature of squamate reptiles. Compared with the group of mammals, a sharp difference was revealed that not only the posterior class Hox genes (PG9 to PG13) but also the central class Hox genes (PG4 to PG8) contained lots of repeats in squamates. According to the collinearity of Hox clusters, the central class Hox genes work co-ordinately to define cervical to lumbar regions during embryonic development by specifying vertebra skeletal regionalization and determining the type of structures (e.g., the different vertebrate ribs) that will form on a given segment (e.g., PG5 and PG6 for rib initiation, PG6 through PG8 for the morphology of the rib cage and PG9 and PG10 for rib suppression [59]). Squamate reptiles exhibit diverse body forms, including the typical four-limbed, pentadactyl, lizard-like morphology, the elongated, limbless, snake-like body form and many intermediate stages of body shapes (with small limbs and reduced digit numbers but not fully limbless). It seemed plausible to associate the frequent appearance of repeats in the central class as well as the posterior class Hox genes with the concurrent highly derived trunk regions of squamate reptiles.

The many amino acid repeats revealed in our study corroborated with the general pattern of tandem repeats previously observed from anole genomic sequences [38], and demonstrated that tandem repeats are also frequent in the coding regions though there are constraints from reading frames and protein structures. Compared with regular sequences, tandem repeats can alter their lengths through repeat number mutations that typically occur at rates orders of magnitude greater than single-nucleotide mutations [60, 61]. Within a protein, amino acid repeats are often embedded in disordered regions and serve as flexible linkers between functional domains that mediate protein–nucleic acid or protein–protein interactions [27]. The expansion and contraction of amino acid repeat may affect the distance between functional domains that would further affect the protein–nucleic acid or protein–protein interactions. Amino acid repeats, thus like the “tuning knobs”, enable fine and relatively safe changes in the protein functions. Some amino acid repeats, e.g., those conserved among orthologous proteins from different species, probably have fixed yet unknown functions; others, especially those appeared recently, can supply the protein interaction network with many potential “tuning knobs”, making the network more flexible for evolutionary adjustment. The presence of numerous amino acid repeats in the green anole genome with about half appearing relatively recently, is probably a way the green anole genome adopted to increase its mutability. Note that we also detected a large number of amino acid repeats in the conserved Hox genes in many squamates, most of which were non-clade common, that is, most of which appeared independently in different lineages. Thus other squamates also seem to tolerate amino acid repeats to some extent and use them as a way to increase their genetic variability. Nevertheless, this does not indicate that the species with low content of amino acid repeat are genetically invariable. Different species may have developed different strategies to make their genomes malleable. In addition to tandem repeats, the anole genome also shows high tolerance for mobile elements that comprise a wide diversity of active mobile element families [37]. Mobile elements can influence the regions that they insert into and are speculated to form substrates for exaptation of novel regulatory elements [62, 63]. The abundant tandem amino acid repeats and a large number of young and active mobile elements are the distinct features of the green anole genome, both of which have the potential to increase the genome’s mutability in many ways.

## Conclusions

In this study we identified an abundance of amino acid repeats in the green anole proteome. Compared with the other vertebrate species in a large orthologous protein data set, the green anole possessed the highest number of amino acid repeats. In addition, these amino acid repeat containing genes were enriched in genes related to transcription and development. It also exhibited the greatest number and largest proportion of amino acid repeats that were not found in the other species, indicating a high tolerance for repeat generation of the anole genome. These data add to our knowledge about the amino acid repeat inventory of vertebrate and indicate that high amino acid repeat content is also found outside mammals in cold-blooded species like lizards. We intensively studied the amino acid repeats in the Hox gene family in a wide collection of squamate species and found an enrichment of amino acid repeat containing Hox genes in the central as well as the posterior class genes in squamates. Squamate reptiles are a diverse clade that has evolved remarkable morphological and physiological adaptations. Genomic studies from different angles can provide clues for understanding the evolutionary diversity of squamates. The enrichment of amino acid repeats found in this study may be one of the mechanisms yet to be investigated to understand the genetic plasticity of the anoles and possibly that of squamates, which have led to impressive evolutionary agility under selection.

### Availability of data

The sequences of the Hox gene fragments identified in this study have been deposited in NCBI GenBank database under accession numbers KC536653 - KC536995.

A few Hox gene fragments did not match the sequence length criterion of NCBI and are available in Additional file 15.

## Additional files

Additional file 1:
**The primers used for amplification of Hox gene fragments.** (DOC 74 kb) Additional file 2:
**The list of all the identified amino acid repeats in the green anole proteome.** (CSV 1122 kb) Additional file 3:
**Statistics of the other amino acid repeat types in the green anole proteome (supplementary table for Table** 2 **).** (DOCX 67 kb) Additional file 4:
**The number of amino acid repeats for each amino acid type at different lengths in the green anole proteome.** (DOC 91 kb) Additional file 5:
**Average spatial distribution of the amino acid repeats in the corresponding proteins.** Only repeats of the commonly found amino acid repeat types were calculated. The spatial distribution was obtained by calculating the ratio of the start point of the repeat to the total length of the repeat containing protein. (PDF 118 kb) Additional file 6:
**The list of all the identified amino acid repeats in the green anole orthologous data set.** (CSV 553 kb) Additional file 7:
**The list of all the identified amino acid repeats in the human orthologous data set.** (CSV 540 kb) Additional file 8:
**The list of all the identified amino acid repeats in the mouse orthologous data set.** (CSV 527 kb) Additional file 9:
**The list of all the identified amino acid repeats in the Chinese softshell turtle orthologous data set.** (CSV 332 kb) Additional file 10:
**The list of all the identified amino acid repeats in the zebra finch orthologous data set.** (CSV 359 kb) Additional file 11:
**The list of all the identified amino acid repeats in the Western clawed frog orthologous data set.** (CSV 286 kb) Additional file 12:
**The commonly found amino acid repeats in the six species.** (DOC 35 kb) Additional file 13:
**The number of rare amino acid repeats in the six species.** (DOC 30 kb) Additional file 14:
**Distribution of amino acid repeat types in proteins from different functional groups.** The functional groups were defined from Gene Ontology annotations (see Methods). For each functional group, the percentage difference of an amino acid type with respect to its distribution in all proteins in the orthologous data set is shown. “Others” includes all the remaining amino acid types besides listed in detail. (PDF 503 kb) Additional file 15:
**The gene identifiers of all the analysed Hox genes.** (CSV 59 kb) Additional file 16:
**The list of identified amino acid repeats in the Hox family.** (CSV 96 kb)

## Abbreviations

AA: amino acid
BLAST: Basic Local Alignment Search Tool
cDNA: complementary DNA
GO: Gene Ontology
PG: paralogous groups
PLP: proportion of the longest consecutive pure codon to the complete repeat size
PRANK: The Probabilistic Alignment Kit
RCP: repeat-containing proteins
UCSC: University of California, Santa Cruz
