# Supplementary material for: Tandem amino acid repeats in the green anole (Anolis carolinensis) and other squamates may have a role in increasing genetic variability
Source: BMC Genomics. 2016 Feb 12;17:109. doi: 10.1186/s12864-016-2430-y (PMC4751654; doi:10.1186/s12864-016-2430-y)
Supplement: Additional file 1: — The primers used for amplification of Hox gene fragments. (DOC 74 kb) [file 12864_2016_2430_MOESM1_ESM.doc]

**Additional file 1 - The primers used for amplification of Hox gene fragments**

| Target gene | Forward primer | | Reverse primer | | Product length(bp) | Notes |
| --- | --- | --- | --- | --- | --- | --- |
|  | Primer name | Sequence | Primer name | Sequence |  |  |
| PG2 | HoxA2_5’_338F | CCATACGGCYGTAATCAGTGAAT | Hox2S_E1_300R* | YTTYTTCTCYTTCATCCANGG | 500-600 | - |
|  | Hox2S_E1_10F* | GAATTYGAGMGRGARATHGGNTT |  |  | 280-370 | all |
| PG3 | Hox3S_E1_1F* | ATGCARAAARCRRCNTAYTAYGA | Hox3S_E1_486R* | TCYTTCATCCANGGRAADATNTG | 400-480 | all |
|  | HoxB3_E1_1F | ATGCAGAAARCRRCNTACTACGA |  |  | 400-480 | A3,D3 |
|  | HoxD3_E1_1F* | ATGCAGAAARCNGCBTAYTAYGA |  |  | 400-480 | A3,B3 |
| PG4 | HoxA4_5'_397F | AATTAGTATTTTTGCACTTCACAA | HoxAD4_E1_750R | AYYTTCTTCATCCANGGRTANAC | 450-650 | D4 |
|  | HoxB4_5'_103F | AACTTTGTTCACTTGACAGTAAGTAGG | HoxB4_I_582R | GYMATAAAAATTTATGGRGGATGTAATTA | 630-750 | 2nd nested PCR |
|  | HoxC4_5'_328F | AGAAAAACGACAAAGCGAGAAAAATTA | HoxC4_I_968R | TCGTAAATCCMGYTCAATTGTGCTAA | 560-730 | - |
|  | HoxD4_5'_1264F | TATTCAGTTGACAGCAAGTAGGAG | HoxAD4_E1_750R | AYYTTCTTCATCCANGGRTANAC | 490-520 | B4,C4 |
| PG5 | HoxA5_5'_360F | TCATCATAAATTGTGCAAGGGTG | HoxAB5_E1_710R | ATGTGMAGYTTYCKCATCCANGG | 630-810 | - |
|  | HoxB5_5'_150F | CATATTTGGCCGCATACATAGCA |  |  | 670-720 | - |
|  | HoxC5_5'_165F | ATCYCCACCTATAAATTSTSCACT | HoxC5_I_1010R | TTTAGTGGACAGTTTTACGAGC | 700-870 | - |
| PG6 | HoxA6_5'_370F | CCAGATGTACTAATACACAACAAATC | HoxAB6_E1_420R* | GARTTCATCCKYTGCATCCANGG | 490-500 | - |
|  | HoxB6_5'_534F* | AWACTRCTAATAGCTAAASCRCT |  |  | 500 | - |
|  | HoxC6_5'_230F | CTTTGTCATTTTGTCTGTCCTGGA | HoxC6_I_785R | CRTAAAGVHRGTTCARTTTATTTAATTTAT | 550 | - |
| PG7 | HoxA7_5'_235F | GCYRCCTYGTAAAACCGACAC | Hox7S_E1_380R* | CATCCARGGGTADATNCGRAA | 430-450 | - |
|  | HoxB7_5'_F72* | CTCGTAAAACCGACACTAAAACG |  |  | 440-460 | - |
| PG8 | HoxB8_5'_630F | ATCACNGAAAACTGTTTATGAACTG | HoxB8_I_1940R | TCAGGGTAATTAARCKATAAAGC | 900-1200 | - |
|  | HoxBC8_E1_1F | ATGAGYTCYTAYTTYGTNAAYYC | HoxC8_E1_435R | GTGNGGTCTCATCCANGGRAACAT | 430 | - |
|  | HoxD8_E1_270F | GGATACGATAAYTTRCARAGNCA | HoxD8_E1_430R | TTGWGRTCTCATCCANGGRAACAT | 160 | - |
| PG9 | HoxA9_5'_-180F | ACCAAGTTGTKACRTGAAAYCYGC | HoxA9_E1_420R | AGCGGTTCAGGYTTDATNCCRTA | 450-600 | - |
|  | HoxB9_5'_260F | CTGACGCTTTATCAGSCWSWCGRAA | HoxB9_E1_1080R | CTSYYTTTGTCYTCGCTTCCYTC | 700-900 | - |
|  | HoxC9_5'_730F | GAGAAGATAAAAAGTACAYATACAS | HoxC9_E1_1360R | TCSGNCTTYTCYTCTTTGTGYTT | 600 | - |
|  | HoxD9_E1_10F | AGTGGCACCMTNAGYAAYTAYTA | HoxD9_E1_900R | ACTTACTTGGGTCAAGTTGCTGYTG | 550-1100 | 2nd nested PCR |
| PG10 | HoxA10_5'_-20F | TATYCAGCATGTTKTGCACAAGA | HoxA10_E1_1060R | GGSGCSGGRGASAGYTCYTCNGC | 800-1000 | - |
|  | HoxC10_5'_-30F | TAGTATTCCTCTTTAAAATCCCTC | HoxC10_E1_860RN | TTTSGCTTCRTTGTCNGARTTRTC | 780-880 | - |
|  | HoxD10_5'_-50F | TAGMGATGTCAGCCTACAAAGGA | HoxD10_I_860R | CCCAACTTYGYAAKSATACCGACC | 800-900 | - |
| PG11 | HoxA11_5'_-20F | ATGTTAAGCTCAGCTAMTGCGGA | HoxA11_I_960R | CCTTATATGCTTATAAAACAGCA | 720-900 | - |
|  | HoxC11_5'_-100F | ACTAAGACGGATNGCGCGTCATC | HoxC11_E1_1560R | GAATGCTTATAAAACTCCACAT | 775-1050 | - |
|  | HoxD11_5'_-200F | ACCMCYMGAATCRATCAAGATG | HoxD11_E1_600R | GTARAACTGGTCRAAGCCYTGNGG | 570-810 | - |
| PG12 | HoxC12_5'_-20F | AASTAGAAGCTCTTGGTCAGG | HoxC12_I_910R | GGGATRTTTACATGTTTATAGG | 800-920 | 2nd nested PCR |
| PG13 | HoxA13_5'_-30F | GAGCCACTRGTCTCAATGCRGATC | HoxA13_E1_980R | GGSAGRGNSGAYTTCCASAGGTG | 650-720 | - |
|  | HoxC13_5'E1_-6F | CATGHTATGACGACTTCNCT | HoxC13_E1_1040R | CTGGGAARGGBGAYTTCCAGARRTG | 660-700 | - |
|  | HoxD13_E1_70F | GGSCAGTGCCGNAAYTTYCTCTC | HoxD13_E1_880R | ACCTGGRAAGGANGAYTTCCARAA | 550-700 | - |
| All paralog group but PG2 | | | HoxPG3-13_E2_R | TTCATNCKNCKRTTYTGRAACCADAT |  | 1st nested PCR |

Notes: PG: paralog group; Primer name: target gene name_location of the primer (5’: 5’ UTR; E1: coding region of exon one; I: intron)_location in our alignments (-200 means that the primer is located 200 bp upstream of the start codon); primers marked with ‘*’ are from our previous publication (Liang et al. 2011); Notes: ‘all’ means that this pair of primers can be used to amplify all the members of the paralog group; gene name indicates that this pair of primers can also yield other gene of the paralog group; 1st nested PCR and 2nd nested PCR denote the reverse primers used in the 1st round and 2nd round of PCR when semi nested PCR is applied.
