# Supplementary material for: Tandem amino acid repeats in the green anole (Anolis carolinensis) and other squamates may have a role in increasing genetic variability
Source: BMC Genomics. 2016 Feb 12;17:109. doi: 10.1186/s12864-016-2430-y (PMC4751654; doi:10.1186/s12864-016-2430-y)
Supplement: Additional file 3: — Statistics of the other amino acid repeat types in the green anole proteome (supplementary table for Table 2 ). (DOCX 67 kb) [file 12864_2016_2430_MOESM3_ESM.docx]

Additional file 3 - Statistics of the other amino acid repeat types in the green anole proteome (supplementary table for Table 2)

| AA type | Count | Average length (Maximum length) | Average length of consecutive AA tract (Maximum length) | Average PLP | Number of PLP=1 (Percentage) | Number of Codons PLP=1 |
| --- | --- | --- | --- | --- | --- | --- |
| R | 111 | 7 (13) | 5.5 (11) | 0.42 | 7 (6.3%) | AGG (4), AGA (1), CGG (1), CGC (1) |
| T | 90 | 10.5 (29) | 5.9 (12) | 0.42 | 7 (7.8%) | ACC (6), ACT (1) |
| H | 74 | 11 (26) | 6.7 (17) | 0.45 | 10 (13.5%) | CAC (8), CAT (2) |
| N | 55 | 8.7 (18) | 7.7 (18) | 0.79 | 23 (41.8%) | AAT (14), AAC (9) |
| I | 48 | 9.6 (24) | 7.9 (21) | 0.68 | 14 (29.2%) | ATT (9), ATA (5) |
| V | 39 | 7 (17) | 5.7 (13) | 0.62 | 9 (23%) | GTT (4), GTG (3), GTC (1), GTA (1) |
| C | 18 | 7.7 (14) | 5.8 (9) | 0.64 | 6 (33.3%) | TGC (4), TGT (2) |
| Y | 17 | 10.4 (18) | 8.6 (18) | 0.74 | 8 (47%) | TAT (8) |
| F | 15 | 6.1 (10) | 5.3 (7) | 0.63 | 3 (20%) | TTC (2), TTT (1) |
| M | 2 | 5 (5) | 5 (5) | 1 | 2 (100%) | ATG (2) |

All lengths in this table refer to number of amino acid. AA means amino acid. PLP is the proportion of the longest consecutive pure codon run size to the complete repeat size.
