# Supplementary material for: Tandem amino acid repeats in the green anole (Anolis carolinensis) and other squamates may have a role in increasing genetic variability
Source: BMC Genomics. 2016 Feb 12;17:109. doi: 10.1186/s12864-016-2430-y (PMC4751654; doi:10.1186/s12864-016-2430-y)
Supplement: Additional file 4: — The number of amino acid repeats for each amino acid type at different lengths in the green anole proteome. (DOC 91 kb) [file 12864_2016_2430_MOESM4_ESM.doc]

**Additional file 4 -** **The number of repeats of each amino acid type at different lengths in the green anole**

| Amino acid type | E | S | P | G | A | L | Q | K | D | R | T | H | N | I | V | C | Y | F | M |
| --- | --- | --- | --- | --- | --- | --- | --- | --- | --- | --- | --- | --- | --- | --- | --- | --- | --- | --- | --- |
| Total repeat number | 1004 | 792 | 686 | 566 | 392 | 361 | 303 | 242 | 138 | 111 | 90 | 74 | 55 | 48 | 39 | 18 | 17 | 15 | 2 |
| Percentage (%) | 20.3 | 16.0 | 13.9 | 11.4 | 7.9 | 7.3 | 6.1 | 4.9 | 2.8 | 2.2 | 1.8 | 1.5 | 1.1 | 1.0 | 0.8 | 0.4 | 0.3 | 0.3 | 0.0 |
| Length=5 | 192 | 172 | 118 | 81 | 99 | 106 | 63 | 66 | 38 | 50 | 25 | 14 | 19 | 11 | 16 | 4 | 2 | 10 | 2 |
| Length=6 | 82 | 51 | 49 | 35 | 35 | 24 | 31 | 16 | 16 | 9 | 6 | 7 | 5 | 3 | 8 | 4 | 2 | 1 |  |
| Length=7 | 102 | 99 | 57 | 57 | 39 | 48 | 37 | 30 | 19 | 14 | 9 | 4 | 4 | 5 | 5 | 3 | 1 | 1 |  |
| Length=8 | 95 | 71 | 77 | 69 | 40 | 44 | 35 | 23 | 14 | 6 | 5 | 6 | 5 | 7 | 3 | 2 | 3 | 1 |  |
| Length=9 | 85 | 66 | 50 | 39 | 36 | 31 | 32 | 14 | 12 | 10 | 9 | 1 | 4 | 5 |  | 1 | 2 |  |  |
| Length=10 | 92 | 88 | 70 | 44 | 42 | 37 | 18 | 31 | 14 | 11 | 4 | 7 | 2 | 3 | 1 | 1 |  | 2 |  |
| Length=11 | 62 | 52 | 50 | 40 | 25 | 26 | 14 | 17 | 10 | 8 | 6 | 5 | 1 | 3 | 3 |  | 1 |  |  |
| Length=12 | 39 | 28 | 31 | 29 | 14 | 12 | 12 | 14 | 2 | 2 | 4 | 5 | 4 | 1 | 1 | 2 |  |  |  |
| Length=13 | 24 | 25 | 17 | 25 | 9 | 7 | 9 | 11 | 3 | 1 | 3 | 6 | 4 | 1 | 1 |  | 1 |  |  |
| Length=14 | 25 | 24 | 41 | 29 | 10 | 9 | 5 | 3 | 3 |  | 1 | 2 |  | 1 |  | 1 | 1 |  |  |
| Length=15 | 38 | 18 | 13 | 17 | 12 | 4 | 8 | 4 | 1 |  | 3 | 4 | 1 | 1 |  |  |  |  |  |
| Length=16 | 15 | 13 | 17 | 15 | 4 | 3 | 4 | 3 |  |  | 1 |  | 2 | 1 |  |  | 2 |  |  |
| Length=17 | 24 | 15 | 21 | 16 | 6 | 3 | 4 | 2 | 1 |  | 2 | 3 | 2 | 1 | 1 |  |  |  |  |
| Length=18 | 21 | 9 | 10 | 8 | 6 | 3 | 5 | 2 |  |  | 1 | 2 | 2 | 1 |  |  | 2 |  |  |
| Length=19 | 12 | 8 | 2 | 7 | 4 | 1 | 5 | 1 |  |  |  | 2 |  |  |  |  |  |  |  |
| Length=20 | 18 | 7 | 11 | 8 | 3 | 1 | 3 | 1 |  |  | 2 | 3 |  | 2 |  |  |  |  |  |
| Length=21 | 12 | 4 | 10 | 12 | 3 |  | 2 |  | 1 |  | 1 |  |  | 1 |  |  |  |  |  |
| Length=22 | 3 | 8 | 3 | 3 | 1 | 1 | 2 |  |  |  | 1 |  |  |  |  |  |  |  |  |
| Length=23 | 7 | 5 | 6 | 1 |  |  | 3 |  | 2 |  |  |  |  |  |  |  |  |  |  |
| Length=24 | 5 | 3 | 3 | 5 | 1 |  |  |  | 1 |  |  |  |  | 1 |  |  |  |  |  |
| Length=25 | 3 | 3 | 2 | 3 |  |  |  | 1 | 1 |  | 2 |  |  |  |  |  |  |  |  |
| Length=26 | 6 | 3 | 1 | 1 | 1 |  | 1 | 1 |  |  |  | 3 |  |  |  |  |  |  |  |
| Length=27 | 6 | 2 | 5 | 2 | 1 |  | 2 | 1 |  |  | 1 |  |  |  |  |  |  |  |  |
| Length=28 | 5 | 3 | 7 | 2 |  |  |  | 1 |  |  |  |  |  |  |  |  |  |  |  |
| Length=29 | 3 |  | 2 | 3 |  | 1 |  |  |  |  | 4 |  |  |  |  |  |  |  |  |
| Length=30 | 1 | 1 |  |  | 1 |  |  |  |  |  |  |  |  |  |  |  |  |  |  |
| Length>30 | 27 | 14 | 13 | 15 |  |  | 8 |  |  |  |  |  |  |  |  |  |  |  |  |
