# Supplementary figures and images for: Tandem amino acid repeats in the green anole (Anolis carolinensis) and other squamates may have a role in increasing genetic variability
Source: BMC Genomics. 2016 Feb 12;17:109. doi: 10.1186/s12864-016-2430-y (PMC4751654; doi:10.1186/s12864-016-2430-y)

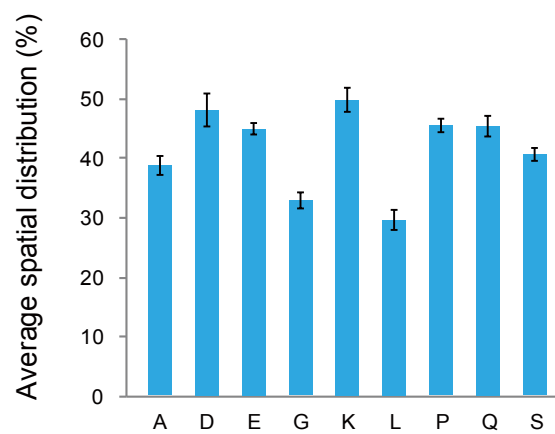

Supplement: Additional file 5: — Average spatial distribution of the amino acid repeats in the corresponding proteins. Only repeats of the commonly found amino acid repeat types were calculated. The spatial distribution was obtained by calculating the ratio of the start point of the repeat to the total length of the repeat containing protein. (PDF 118 kb) [file 12864_2016_2430_MOESM5_ESM.pdf]
