# Supplementary material for: Tandem amino acid repeats in the green anole (Anolis carolinensis) and other squamates may have a role in increasing genetic variability
Source: BMC Genomics. 2016 Feb 12;17:109. doi: 10.1186/s12864-016-2430-y (PMC4751654; doi:10.1186/s12864-016-2430-y)
Supplement: Additional file 12: — The commonly found amino acid repeats in the six species. (DOC 35 kb) [file 12864_2016_2430_MOESM12_ESM.doc]

**Additional file 12 - The commonly found amino acid repeats in the six species**

| Species | 1 | 2 | 3 | 4 | 5 | 6 | 7 | 8 |
| --- | --- | --- | --- | --- | --- | --- | --- | --- |
| Human | E (394) | P (394) | A (345) | S (313) | G (226) | L (208) | Q (152) | K (141) |
| Mouse | E (382) | P (348) | A (303) | S (299) | G (214) | L (171) | Q (151) | K (142) |
| Zebra finch | E (315) | S (256) | P (222) | A (154) | K (137) | Q (121) | L (92) | G (86) |
| Chinese softshell turtle | E (322) | S (249) | P (216) | K (124) | A (106) | G (98) | Q (95) | L (75) |
| Green anole | E (485) | S (383) | P (328) | G (276) | A (192) | Q (170) | L (153) | K (144) |
| Western clawed frog | S (239) | E (187) | P (183) | K (142) | A (92) | L (86) | G (64) | D (62) |

Numbers in parentheses indicate counts of each repeat type in the species.
