# Supplementary material for: Tandem amino acid repeats in the green anole (Anolis carolinensis) and other squamates may have a role in increasing genetic variability
Source: BMC Genomics. 2016 Feb 12;17:109. doi: 10.1186/s12864-016-2430-y (PMC4751654; doi:10.1186/s12864-016-2430-y)
Supplement: Additional file 13: — The number of rare amino acid repeats in the six species. (DOC 30 kb) [file 12864_2016_2430_MOESM13_ESM.doc]

**Additional file 13 - The number of rare amino acid repeats in the six species**

| Amino acid type | Human | Mouse | Zebra finch | Chinese softshell turtle | Green anole | Western clawed frog |
| --- | --- | --- | --- | --- | --- | --- |
| I (Isoleucine) | 3 | 3 | 2 | 2 | 14 | 5 |
| F (Phenylalanine) | 1 | 1 | 2 | 1 | 5 | 3 |
| M (Methionine) | 0 | 0 | 0 | 0 | 1 | 0 |
| N (Asparagine) | 8 | 5 | 5 | 7 | 16 | 12 |
