# Supplementary material for: Tandem amino acid repeats in the green anole (Anolis carolinensis) and other squamates may have a role in increasing genetic variability
Source: BMC Genomics. 2016 Feb 12;17:109. doi: 10.1186/s12864-016-2430-y (PMC4751654; doi:10.1186/s12864-016-2430-y)
Supplement: Additional file 14: — Distribution of amino acid repeat types in proteins from different functional groups. The functional groups were defined from Gene Ontology annotations (see Methods). For each functional group, the percentage difference of an amino acid type with respect to its distribution in all proteins in the orthologous data set is shown. “Others” includes all the remaining amino acid types besides listed in detail. (PDF 503 kb) [file 12864_2016_2430_MOESM14_ESM.pdf]

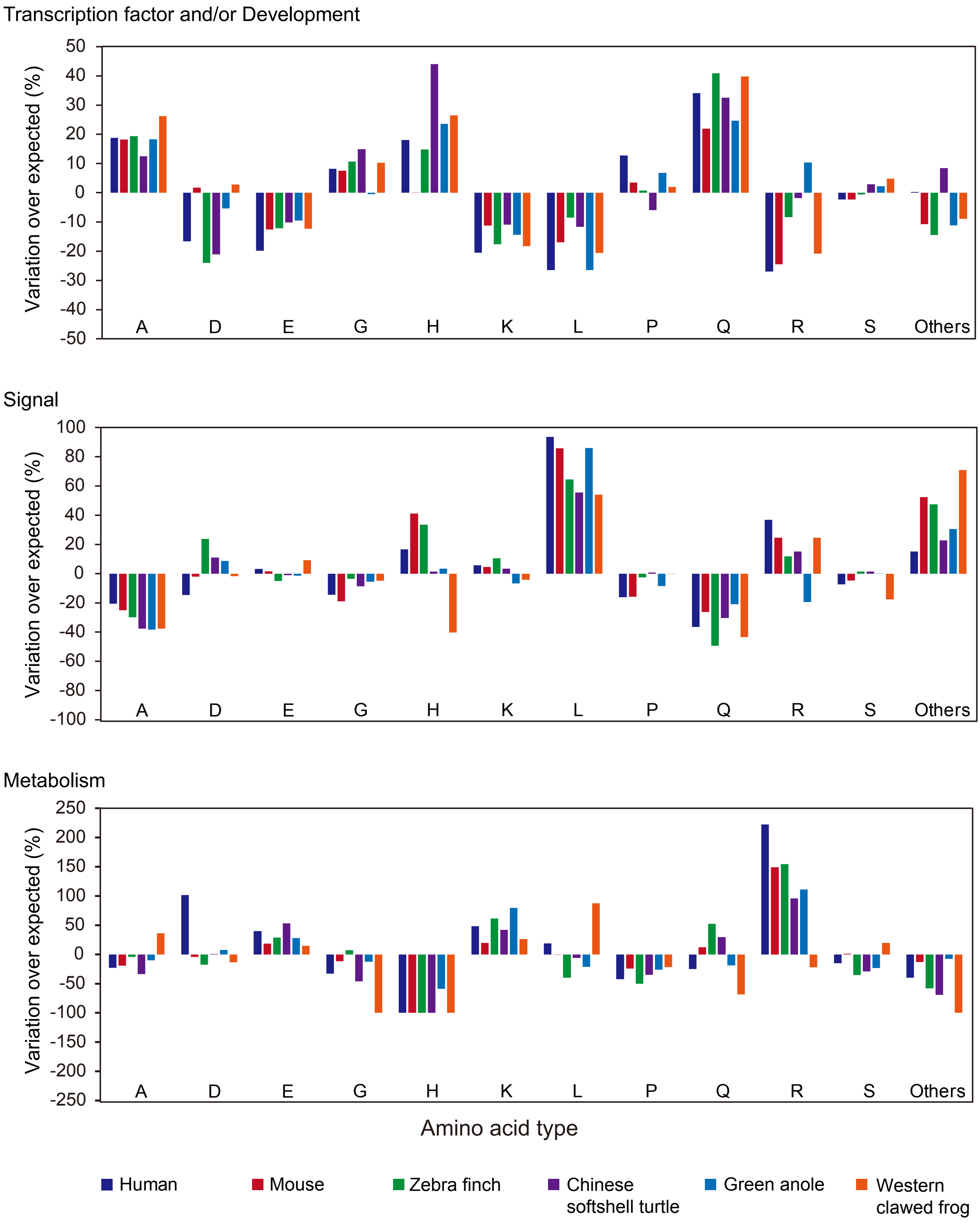

Transcription factor and/or Development
Variation over expected (%)
50
40
30
20
10
0
-10
-20
-30
-40
-50
A
D
E
G
H
K
L
P
Q
R
S
Others
Signal
Variation over expected (%)
100
80
60
40
20
0
-20
-40
-60
-80
-100
A
D
E
G
H
K
L
P
Q
R
S
Others
Metabolism
Variation over expected (%)
250
200
150
100
50
0
-50
-100
-150
-200
-250
A
D
E
G
H
K
L
P
Q
R
S
Others
Amino acid type
Human
Mouse
Zebra finch
Chinese softshell turtle
Green anole
Western clawed frog
